# Plasma p‐tau217 and p‐tau217/Aβ1‐42 are effective biomarkers for identifying CSF‐ and PET imaging‐diagnosed Alzheimer's disease: Insights for research and clinical practice

**DOI:** 10.1002/alz.14536

**Published:** 2025-01-30

**Authors:** Xiaomei Zhong, Qiang Wang, Mingfeng Yang, Gaohong Lin, Kexin Yao, Zhangying Wu, Danyan Xu, Huarong Zhou, Ben Chen, Haishan Shi, Min Zhang, Xiaolei Shi, Yijie Zeng, Jingyi Lao, Shuang Liang, JiaFu Li, Qin Liu, Huanmin Liu, Yunheng Chen, Yicheng Lin, Cong Ouyang, Jieqin Lv, Xiang Liang, Yuwang Cheng, Pengcheng Ran, Baoying Gong, Bin Zhang, Jianwen Guo, Hong Zhang, Sen Liu, Jihui Zhang, Haiying Liu, Yuping Ning

**Affiliations:** ^1^ The Affiliated Brain Hospital Guangzhou Medical University Guangzhou China; ^2^ Departments of Nuclear Medicine The Second Affiliated Hospital of Guangzhou University of Chinese Medicine Guangzhou China; ^3^ State Key Laboratory of Dampness Syndrome of Chinese Medicine The Secondary Hospital of Guangzhou University of Chinese Medicine Guangdong Provincial Hospital of Traditional Chinese Medicine Guangzhou China; ^4^ Pason Neuroscience Research Center Beijing China; ^5^ Key Laboratory of Neurogenetics and Channelopathies of Guangdong Province and the Ministry of Education of China Guangzhou Medical University Guangzhou China

**Keywords:** Alzheimer's disease, amyloid beta, blood biomarkers, clinical practice, mild cognitive impairment, phosphorylated tau217

## Abstract

**INTRODUCTION:**

With the advancement of disease‐modifying therapies for Alzheimer's disease (AD), validating plasma biomarkers against cerebrospinal fluid (CSF) and positron emission tomography (PET) standards is crucial in both research and real‐world settings.

**METHODS:**

We measured plasma phosphorylated tau (p‐tau)217, p‐tau181, amyloid beta (Aβ)1‐40, Aβ1‐42, and neurofilament light chain in research and real‐world cohorts. Participants were categorized by brain amyloid status using US Food and Drug Administration/European Medicines Agency–approved CSF or PET methods.

**RESULTS:**

Plasma p‐tau217 and p‐tau217/Aβ1‐42 demonstrated superior accuracy in detecting brain amyloid pathologies, with area under the curve from 0.94 to 0.97 in all cohorts. Specificity was lower in the real‐world cohort but improved significantly by integrating demographic and clinical factors, aligning performance with research cohorts. Additionally, plasma biomarkers exhibited strong correlations with their CSF counterparts and PET standardized uptake value ratios, with significant associations in amyloid‐positive participants.

**DISCUSSION:**

Plasma p‐tau217 and p‐tau217/Aβ1‐42 are effective diagnostic tools. However, patient demographics, apolipoprotein E ε4 status, and cognitive condition must be considered to improve specificity in the clinical practice.

**Highlights:**

- Plasma phosphorylated tau (p‐tau)217 and p‐tau217/amyloid beta (Aβ)1‐42 demonstrated exceptional accuracy (area under the curve: 0.94–0.97) in detecting brain amyloid pathologies across both research (Southern China Aging Brain Initiative [SCABI]‐1, SCABI‐2) and real‐world clinical practice (RCP) cohorts.
- Incorporating patient‐specific factors (sex, age, apolipoprotein E ε4, cognitive status) improved diagnostic specificity in the clinical RCP cohort, aligning its performance with that of research cohorts.
- Plasma biomarkers, particularly p‐tau217 and their ratios, showed robust correlations with cerebrospinal fluid biomarkers and positron emission tomography amyloid standardized uptake value ratios, underscoring their value as non‐invasive diagnostic alternatives.
- Plasma p‐tau217 and p‐tau217/Aβ1‐42 proved highly effective in diagnosing amyloid burden, offering a practical solution to bridge research advancements with real‐world clinical practice.

## 1 BACKGROUND

Alzheimer's disease (AD) is an age‐related, irreversible neurodegenerative disorder accounting for 60% to 70% of all dementia cases.^1^ It is characterized by the deposition of amyloid beta (Aβ)‐containing plaques in the extracellular space of the brain parenchyma and the formation of intraneuronal tau tangle aggregates.^2^ AD progresses over an extended preclinical phase that lasts 10 to 20 years, during which Aβ plaques accumulate in the cortex, and tau pathology propagates from the medial temporal lobe to neocortical areas.^3^

Anti‐amyloid antibodies can reduce Aβ plaques in the brain, slowing cognitive decline in individuals with mild cognitive impairment (MCI) and AD‐related dementia.^4^, ^5^ However, initiating these therapies requires confirmation of Aβ positivity, typically through positron emission tomography (PET), which allows the visualization of Aβ deposition in the brain or cerebrospinal fluid (CSF) assays and measurement of Aβ1‐42 levels or the Aβ1‐42/Aβ1‐40 ratio, as well as phosphorylated tau (p‐tau) or total tau (t‐tau) levels. These methods, while effective, are expensive and invasive. Therefore, cost‐effective, minimally invasive, and easily interpretable diagnostic tools are essential.

Recent advancements have enabled the detection of biomarkers in plasma, potentially reshaping the AD diagnostic landscape and enabling large‐scale early detection of AD.^6^ Plasma biomarkers such as p‐tau217, p‐tau181, the Aβ1‐42/Aβ1‐40 ratio, and neurofilament light chain (NfL) have shown specificity for AD.^7^, ^8^, ^9^, ^10^, ^11^, ^12^ Despite these advancements, plasma biomarkers have not yet gained widespread acceptance as stand‐alone clinical diagnostic tests.^13^ This is due to the need for replicable studies validating their diagnostic consistency with CSF and PET biomarkers and the lack of globally accepted cutoff values for indicating brain amyloid pathology (concerning CSF or PET findings) in research and clinical practice.

The Lumipulse G system, approved by the US Food and Drug Administration (FDA) and the European Union In Vitro Diagnostics Regulation (EU IVDR), is a fully automated platform that reliably measures the Aβ1‐42/Aβ1‐40 ratio in CSF and can profile AD biomarkers by combining these results with t‐tau and p‐tau181 levels.^14^, ^15^ Recently, several plasma analytes (Aβ1‐42, Aβ1‐40, p‐tau181, p‐tau217, and NfL) have been integrated into the fully automated Lumipulse G system using chemiluminescent enzyme immunoassay technology. Nevertheless, this system lacks sufficient clinical validation data^16^, ^17^ and validated, optimized cutoff values for these plasma biomarkers in identifying the burden of brain amyloid for use in research and clinical practice.

This study aimed to evaluate the diagnostic performance and utility of plasma biomarkers in distinguishing amyloid‐positive from amyloid‐negative individuals across research and real‐world settings. Specifically, the study sought to: (1) compare the ability of various plasma biomarkers to detect brain amyloid pathology, (2) assess differences in diagnostic performance between research and real‐world contexts and evaluate the impact of integrating demographic and clinical factors (e.g., age, sex, apolipoprotein E [*APOE*] ε4 status, and cognitive status) on improving biomarker efficacy in real‐world settings, and (3) examine the correlations between plasma biomarkers and CSF biomarkers or amyloid PET standardized uptake value ratios (SUVRs).

## 2 METHODS

### 2.1 Study cohorts and participants

The Southern China Aging Brain Initiative (SCABI) cohort included 260 voluntary participants recruited from hospitals and the community who underwent 18F‐florbetapir PET scans between March 2021 and June 2024. To facilitate the study objectives, the SCABI cohort was randomly split in a 7:3 ratio into two sub‐cohorts: SCABI‐1, for establishing plasma biomarker cutoffs, and SCABI‐2, for internally validating the cutoffs. Additionally, an independent real‐world clinical practice (RCP) cohort comprising 100 consecutive participants undergoing lumbar puncture was recruited from the Affiliated Brain Hospital of Guangzhou Medical University to externally validate the established cutoffs. All participants or their legal guardians provided signed informed consent. The study adhered to the Declaration of Helsinki and was approved by the ethics committees of the Affiliated Brain Hospital of Guangzhou Medical University.

The SCABI cohort included participants who met the following criteria: (1) ≥ 50 years of age; and (2) cognitively normal, or MCI (according to the Peterson criteria^18^), or dementia (according to the criteria for any dementia according to the Diagnostic and Statistical Manual of Mental Disorders, 4th edition [DSM‐IV]^19^). The exclusion criteria were as follows: (1) malignant tumors and severe cerebrovascular diseases (including ischemic stroke and intracerebral hemorrhage with neurological deficit symptoms), (2) severe neurological disorders (such as metabolic brain diseases, encephalitis, multiple sclerosis, epilepsy, traumatic brain injury, normal pressure hydrocephalus, etc.), (3) severe mental illness (schizophrenia, bipolar affective disorder, schizoaffective disorder, paranoid psychosis, intellectual disability, etc.), and (4) systemic diseases causing cognitive impairment (liver dysfunction, renal insufficiency, thyroid dysfunction, severe anemia, folate or vitamin B12 deficiency, syphilis, human immunodeficiency virus infection, alcohol, and drug abuse, etc.).

The RCP cohort consisted of consecutive participants who underwent lumbar puncture (LP) at the hospital as part of routine clinical care. Inclusion criteria required participants to be cognitively normal, have MCI (based on Petersen criteria^18^), or have dementia (according to DSM‐IV criteria for any dementia^19^). LPs were performed for several clinical indications, including the differential diagnosis of dementia, confirmation of AD pathology in suspected cases, and investigation of unexplained cognitive symptoms. These procedures were ordered by neurologists based on patient symptoms and were part of routine diagnostic workflows rather than research‐specific protocols. Participants provided informed consent for the use of their clinical and CSF data in the study. The RCP cohort reflects a real‐world clinical population, which differentiates the RCP cohort from the SCABI cohort, which represents a more controlled research environment, and provides valuable insights into the utility of plasma biomarkers in diverse clinical contexts.

**RESEARCH IN CONTEXT**

1. **Systematic review**: A comprehensive PubMed review identified recent advancements in blood‐based biomarkers for Alzheimer's disease (AD), particularly amyloid beta (Aβ) and phosphorylated tau (p‐tau). While numerous studies have explored these biomarkers, few have directly compared their performance in research and real‐world clinical settings. Relevant studies are cited throughout the article to contextualize this work.
2. **Interpretation**: Plasma p‐tau217 and p‐tau217/Aβ1‐42 exhibited excellent diagnostic accuracy for amyloid positivity (area under the curve: 0.94–0.97) across research (Southern China Aging Brain Initiative [SCABI]‐1, SCABI‐2) and real‐world clinical practice (RCP) cohorts. Specificity was lower in the real‐world RCP cohort but improved significantly by integrating demographic and clinical factors (e.g., age, sex, apolipoprotein E ε4, cognitive status), aligning performance with research cohorts. Plasma p‐tau217 and its ratios correlated strongly with cerebrospinal fluid biomarkers and positron emission tomography amyloid standardized uptake value ratios (*R* ^2^ > 0.4, *P* < 0.001) confirming their potential as non‐invasive diagnostic tools.
3. **Future directions**: Our findings support the integration of plasma biomarkers, particularly p‐tau217 and p‐tau217/Aβ1‐42, as diagnostic tools in clinical practice. However, further studies are needed to evaluate their utility in longitudinal assessments, accounting for patient‐specific factors that could influence accuracy. Additionally, future research should explore the role of other plasma biomarkers, such as neurofilament light chain and p‐tau181, and validate these findings in diverse populations.

AD was diagnosed according to the criteria for dementia due to AD in the diagnostic guidelines for probable AD developed by the National Institute on Aging–Alzheimer's Association workgroups^20^ and further confirmed via positive Aβ PET findings or positive CSF Aβ1‐42/Aβ1‐40 ratios.

### 2.2 Cognitive evaluation

As previously described, participants underwent a comprehensive neuropsychological evaluation using sensitive tests assessing all cognitive domains:^21^ the Mini‐Mental State Examination (MMSE), Clinical Dementia Rating (CDR) scale, activities of daily living (ADL) scale, Hachinski Ischemic Score (HIS), Auditory Verbal Learning Task (AVLT), Trail‐Making Test (TMT), Symbol Digit Modalities Test (SDMT), Boston Naming Test (BNT), Rey‒Osterrieth Complex Figure (ROCF) test, Stroop Color and Word Test (SCWT), Digit Span Test (DST), and Clock Drawing Test (CDT).

### 2.3 CSF sample acquisition and analysis

CSF samples were collected from 100 participants using 5 mL polypropylene tubes and processed within a 4 hour window. After centrifugation (2000 × g for 10 minutes at 4°C), aliquots of 0.5 mL were stored at –80°C until further analysis. The CSF Aβ1‐42/Aβ1‐40 ratio and p‐tau, t‐tau, and NfL levels were determined using the automated immunoassay analyzer Lumipulse G1200 (Fujirebio). This was done with the aid of CSF kits such as Lumipulse G β‐Amyloid 1‐40, Lumipulse G β‐Amyloid 1‐42, Lumipulse G pTau 181, Lumipulse G Total Tau, and Lumipulse G NfL CSF. As per FDA recommendations, a CSF Aβ1‐42/Aβ1‐40 ratio of 0.059 is the cutoff for differentiating between amyloid‐negative and amyloid‐positive brain states.^14^

### 2.4 Plasma sample acquisition and analysis

Plasma samples were collected from 360 participants into 5 mL polypropylene tubes and promptly transported to the laboratory for processing within 4 hours of collection. After centrifugation at 2000 × g for 10 minutes at 4°C, aliquots of 0.5 mL plasma were transferred into separate polypropylene tubes and stored at –80°C until needed for analysis. The time frame for plasma collection was within 3 months of the PET scan or CSF collection.

The plasma samples were thawed at room temperature on the day of analysis. To avoid the impact of multiple freeze–thaw cycles, only aliquots that had not been previously thawed were used in this study. The levels of plasma Aβ1‐42, Aβ1‐40, NfL, p‐tau181, and p‐tau217 were measured directly from the storage tubes containing 0.5 mL of plasma. This was done using the Lumipulse G β‐Amyloid 1‐40 Plasma, Lumipulse G β‐Amyloid 1‐42 Plasma, Lumipulse G pTau 181 Plasma, Lumipulse G pTau 217 Plasma, and Lumipulse G NfL Blood assays on the Lumipulse G1200 automated platform (Fujirebio). The manufacturer's instructions carried out all testing procedures. This included vortexing and centrifugation after thawing to prevent fibrin clots from influencing the results.

### 2.5 *APOE* status

The Lumipulse G ApoE4 and Pan‐ApoE assays (Fujirebio) were used sequentially to analyze *APOE* ε4 and Pan‐*APOE*, respectively. The *APOE* proteotype status, which indicates the presence of *APOE* ε4 alone (homozygous) or in combination with *APOE* ε2 or *APOE* ε3 (heterozygous) in human plasma, was determined by calculating the *APOE* ε4/Pan‐*APOE* ratio. The samples were classified as follows: “null” (indicating the absence of *APOE* ε4) if the ratio was < 5%, “heterozygous” (indicating the presence of *APOE* ε4 with either *APOE* ε2 or *APOE* ε3) if the ratio was ≥ 5% but < 75%, and “homozygous” (indicating the presence of *APOE* ε4 without either *APOE* ε2 or *APOE* ε3) if the ratio was ≥ 75%.

### 2.6 Amyloid PET imaging acquisition, visual assessment, and quantitative analysis

A total of 260 participants underwent amyloid PET imaging with 18F‐florbetapir. The PET data were captured using a SIGNA PET magnetic resonance (MR) device or a Siemens PET computed tomography (CT) device 50 minutes after an injection of 10 ± 1 mCi of 18F‐florbetapir.

PET MR images were obtained over 15 minutes, using the ordered subset expectation maximization reconstruction algorithm with time‐of‐flight and point spread function corrections. This process involved 28 subsets and six iterations. The PET images were corrected for attenuation using zero‐echo time sequences. The matrix size was 256 × 256, with a display field of view of 46.2 cm × 30 cm, a slice thickness of 2.78 mm, and a pixel size of 2.8 mm × 2.8 mm.

PET CT scans were acquired over 15 to 20 minutes using a 3D iterative algorithm (4 iterations and 21 subsets) with a matrix size of 336 × 336, a zoom factor of 2.0 mm, a slice thickness of 2.0 mm, and an all‐pass filter. Time‐of‐flight and point spread function algorithms were used with five iterations and 16 subsets for PET image reconstruction. The matrix size was 336 × 336, with a zoom factor of 2.0, a slice thickness of 2.0 mm, and a Gaussian filter with a full width at half‐maximum of 5.0.

Blinded to the participant's clinical diagnoses and biomarker levels, expert readers visually rated all the PET scans. Following FDA recommendations,^22^ scans were classified as “positive” when one or more areas showed increased cortical gray matter signaling, leading to reduced or absent contrast between gray and white matter. Scans were classified as “negative” when the contrast between gray and white matter was clear.

Subsequently, amyloid depositions within the PET scans were quantified using Statistical Parametric Mapping (SPM12, http://www.fil.ion.ucl. ac.uk/spm/; RRID:SCR_007037). The raw PET and T1 images were first converted from DICOM to NIfTI format and underwent origin correction for proper alignment. Nine participants were excluded from the analysis due to contraindications for MR imaging, which resulted in the absence of T1‐weighted images necessary for spatial normalization and subsequent quantification. Spatial normalization was performed by co‐registering the PET images with the corresponding T1‐weighted MR images. The individual MR images were then normalized to the Montreal Neurological Institute 152 template space, and the resulting transformation matrix was applied to the PET images to achieve spatial standardization. A Gaussian smoothing kernel (8 mm full width at half‐maximum) was applied to enhance the signal‐to‐noise ratio. The mean 18F‐florbetapir uptake was calculated across predefined regions of interest, including the frontal cortex, lateral parietal cortex, and anterior/posterior cingulate cortices. Finally, the 18F‐florbetapir SUVR map was generated using the whole gray cerebellum as the reference region, enabling precise quantification of amyloid burden in the brain.

### 2.7 Statistical analysis

#### 2.7.1 Data preprocessing

To make full use of the available data, missing values (refer to Tables S1–S2 in supporting information for missing rates) were imputed via multiple imputation by chained equations (MICE), using the “mice” package (version 3.16.0). Outliers were detected using the Tukey method, which identifies values beyond 1.5 times the interquartile range (IQR), executed with the “outliers” package (version 3). Identified outliers were replaced with their respective quartile values (Q1 or Q3) to minimize their impact on subsequent analyses while preserving the overall data distribution.

#### 2.7.2 Data analysis

Independent samples *t* tests were used to evaluate differences in continuous variables between amyloid‐positive and amyloid‐negative groups, while chi‐square tests were used for categorical variables. The correlation between plasma marker levels and CSF marker levels or PET measurements was assessed using the Pearson test and a linear regression model. Diagnostic performance was evaluated via receiver operating characteristic (ROC) curve analyses to calculate area under the curve (AUC) values with 95% confidence intervals (CIs). Value retention (VR) was calculated to assess overall diagnostic balance, defined as the geometric mean of sensitivity and specificity.

Optimal cutoff values for plasma biomarkers were established using the Youden index in the SCABI‐1 cohort and validated in the SCABI‐2 and RCP cohorts. Sensitivity, specificity, positive percentage agreement (PPA), negative percentage agreement (NPA), and F1 scores were computed alongside confusion matrices to assess classification performance. Logistic regression models incorporating additional variables, including sex, age, *APOE* ε4, and cognitive status, were used to test improvements in real‐world diagnostic balance. The mean differences in each cohort between the AUCs and 95% CIs were compared among the biomarkers using the DeLong test. Statistical analyses were conducted using R (version 4.3.2), with visualizations generated using ggplot2. Statistical significance was defined at α = 0.05, and all tests were two tailed.

## 3 RESULTS

### 3.1 Characterization of the study population

Table 1 presents each cohort's demographic, clinical, cognitive, genetic, and biomarker characteristics based on amyloid status. Across all cohorts, amyloid‐positive participants show significantly higher rates of dementia (SCABI‐1: 30.3%, SCABI‐2: 45.2%, RCP: 81.6%, *P* < 0.05) and lower rates of cognitively unimpaired status compared to amyloid‐negative participants. Amyloid‐positive individuals have higher proportions of *APOE* ε4 carriers, with significant differences observed in all cohorts (e.g., SCABI‐1: 38 .2% vs. 17%, *P* = 0.002).

**TABLE 1:** Clinical and demographic characteristics of all participants based on amyloid status.

	SCABI‐1 cohort (70%)					SCABI‐2 cohort (30%)				RCP cohort			
	[ALL]	Negative	Positive			[ALL]	Negative	Positive		[ALL]	Negative	Positive	
	*N = 182*	*N = 106*	*N = 76*	p.overall		*N* = 78	*N* = 47	*N* = 31	p.overall	*N = 100*	*N = 51*	*N = 49*	p.overall
Cognitive status				<0.001					0.002				0.035
Cognitively unimpaired (%)	36 (19.8%)	31 (29.2%)	5 (6.58%)			18 (23.1%)	14 (29.8%)	4 (12.9%)		1 (1.00%)	1 (1.96%)	0 (0.00%)	
MCI (%)	120 (65.9%)	72 (67.9%)	48 (63.2%)			41 (52.6%)	28 (59.6%)	13 (41.9%)		28 (28.0%)	19 (37.3%)	9 (18.4%)	
Dementia (%)	26 (14.3%)	3 (2.83%)	23 (30.3%)			19 (24.4%)	5 (10.6%)	14 (45.2%)		71 (71.0%)	31 (60.8%)	40 (81.6%)	
Sex (Female %)	121 (66.5%)	71 (67.0%)	50 (65.8%)	0.993		59 (75.6%)	36 (76.6%)	23 (74.2%)	1.000	57 (57.0%)	22 (43.1%)	35 (71.4%)	0.008
*APOE* ε4 (Carrier %)	47 (25.8%)	18 (17.0%)	29 (38.2%)	0.002		23 (29.5%)	10 (21.3%)	13 (41.9%)	0.088	38 (38.0%)	8 (15.7%)	30 (61.2%)	< 0.001
Age	69.0 (7.03)	69.0 (6.63)	69.0 (7.59)	0.970		67.9 (7.58)	67.0 (7.35)	69.2 (7.87)	0.237	65.4 (10.9)	63.2 (10.9)	67.6 (10.6)	0.040
Education	10.1 (4.04)	11.0 (3.80)	8.81 (4.04)	< 0.001		9.77 (4.23)	10.6 (4.21)	8.50 (4.02)	0.034	8.65 (3.70)	9.00 (4.14)	8.33 (3.25)	0.410
MMSE	20.7 (6.41)	23.4 (4.04)	16.9 (7.18)	< 0.001		19.2 (6.39)	21.6 (5.01)	15.5 (6.58)	< 0.001	13.2 (7.66)	15.1 (7.96)	11.5 (7.02)	0.032
ADL	12.8 (2.38)	13.7 (1.02)	11.7 (3.15)	< 0.001		12.7 (2.36)	13.0 (2.27)	12.4 (2.49)	0.291	8.81 (4.56)	9.48 (4.56)	8.20 (4.52)	0.190
HIS	1.76 (0.98)	1.72 (0.93)	1.83 (1.05)	0.459		2.00 (1.08)	2.00 (0.96)	2.00 (1.26)	1.000	2.35 (1.58)	2.19 (1.55)	2.50 (1.62)	0.352
CDR	0.66 (0.50)	0.50 (0.25)	0.89 (0.65)	< 0.001		0.71 (0.54)	0.56 (0.42)	0.94 (0.62)	0.005	1.40 (0.87)	1.27 (0.88)	1.51 (0.87)	0.206
Hypertension	62 (34.1%)	36 (34.0%)	26 (34.2%)	1.000		27 (34.6%)	13 (27.7%)	14 (45.2%)	0.178	27 (27.0%)	10 (19.6%)	17 (34.7%)	0.141
Cardiology	25 (13.7%)	17 (16.0%)	8 (10.5%)	0.397		14 (17.9%)	7 (14.9%)	7 (22.6%)	0.573	10 (10.0%)	7 (13.7%)	3 (6.12%)	0.319
Diabetes mellitus	28 (15.4%)	20 (18.9%)	8 (10.5%)	0.184		9 (11.5%)	5 (10.6%)	4 (12.9%)	1.000	18 (18.0%)	8 (15.7%)	10 (20.4%)	0.723
Dyslipidemia	58 (31.9%)	39 (36.8%)	19 (25.0%)	0.128		27 (34.6%)	16 (34.0%)	11 (35.5%)	1.000	16 (16.0%)	7 (13.7%)	9 (18.4%)	0.719
**Plasma biomarkers**													
Aβ40(pg/ mL)	272 (41.0)	276 (40.8)	266 (40.9)	0.089		263 (45.2)	263 (46.3)	263 (44.2)	0.972	269 (46.1)	265 (48.2)	273 (44.0)	0.391
Aβ42(pg/ mL)	24.6 (4.86)	26.8 (4.53)	21.6 (3.56)	< 0.001		24.7 (4.99)	26.8 (4.25)	21.5 (4.35)	< 0.001	24.4 (5.58)	26.3 (5.34)	22.4 (5.15)	< 0.001
Aβ42/Aβ40	0.09 (0.01)	0.10 (0.01)	0.08 (0.01)	< 0.001		0.09 (0.02)	0.10 (0.02)	0.08 (0.01)	< 0.001	0.09 (0.02)	0.10 (0.02)	0.08 (0.02)	< 0.001
NfL(pg/ mL)	21.6 (7.70)	19.4 (6.82)	24.5 (7.92)	< 0.001		20.4 (7.60)	18.9 (6.20)	22.8 (8.92)	0.038	31.6 (17.3)	32.3 (20.1)	30.8 (14.0)	0.662
ptau181(pg/ mL)	2.98 (1.40)	2.36 (0.98)	3.85 (1.43)	< 0.001		2.84 (1.18)	2.40 (0.90)	3.50 (1.26)	< 0.001	2.74 (1.09)	2.19 (0.86)	3.31 (1.01)	< 0.001
p‐tau217(pg/ mL)	0.38 (0.40)	0.15 (0.06)	0.70 (0.46)	< 0.001		0.39 (0.42)	0.15 (0.05)	0.75 (0.48)	< 0.001	0.48 (0.38)	0.21 (0.12)	0.76 (0.35)	< 0.001
**SUVRs (only in SCABI)**	1.17 (0.19)	1.06 (0.11)	1.32 (0.18)	< 0.001		1.18 (0.21)	1.07 (0.12)	1.35 (0.21)	< 0.001				
**CSF biomarkers (only in RCP)**													
Aβ40(pg/ mL)	–	–	–	–	–	–	–	–	–	5993 (2935)	5697 (3346)	6300 (2432)	0.305
Aβ42(pg/ mL)	–	–	–	–	–	–	–	–	–	361 (242)	458 (290)	260 (113)	< 0.001
Aβ42/Aβ40	–	–	–	–	–	–	–	–	–	0.06 (0.02)	0.08 (0.02)	0.04 (0.01)	< 0.001
t‐tau(pg/ mL)	‐	–	‐	–	‐	–	‐	–	‐	479 (273)	311 (160)	653 (258)	< 0.001
p‐tau181(pg/ mL)	–	–	–	–	–	–	–	–	–	75.7 (61.4)	38.0 (29.1)	115 (61.6)	< 0.001
NfL(pg/ mL)	–	–	–	–	–	–	–	–	–	1334 (1266)	1484 (1608)	1177 (750)	0.222

Abbreviations: ADL, activities of daily living; Aβ, amyloid beta; *APOE*, apolipoprotein E; CDR, Clinical Dementia Rating; CSF, cerebrospinal fluid; HIS, Hachinski Ischemic Score; MCI, mild cognitive impairment; MMSE, Mini‐Mental State Examination; NfL, neurofilament light chain; RCP, real‐world clinical practice; SCABI, Southern China Aging Brain Initiative; SUVR, standardized uptake value ratio; p‐tau, phosphorylated tau; t‐tau, total tau.

Mean age does not differ significantly between amyloid‐positive and amyloid‐negative participants in the SCABI‐1 and SCABI‐2 cohorts, but a small significant difference is seen in the RCP cohort (67.6 vs. 63.2 years, *P* = 0.04). Amyloid‐positive individuals have lower MMSE scores in all cohorts (e.g., SCABI‐1: 16.9 vs. 23.4, *P* < 0.001). ADL scores indicate greater functional impairment in amyloid‐positive participants, though significance is observed only in SCABI‐1 (*P* < 0.001). Amyloid‐positive individuals exhibit significantly higher CDR scores across cohorts (e.g., SCABI‐1: 0.89 vs. 0.50, *P* < 0.001).

For plasma biomarkers, amyloid‐positive individuals consistently exhibit lower plasma Aβ1‐42 levels and Aβ1‐42/Aβ1‐40 ratios across all cohorts (*P* < 0.001), indicating reduced amyloid clearance. Plasma p‐tau181 and p‐tau217 levels are markedly higher in amyloid‐positive participants across all cohorts (*P* < 0.001). Elevated plasma NfL levels are observed in amyloid‐positive individuals in SCABI‐1 and SCABI‐2 cohorts (*P* < 0.05), though differences are not significant in the RCP cohort.

Amyloid‐positive individuals display significantly higher SUVRs in both SCABI‐1 and SCABI‐2 cohorts (*P* < 0.001), confirming increased amyloid deposition in the brain. CSF Aβ1‐42 and Aβ1‐42/Aβ1‐40 ratio are significantly lower in amyloid‐positive individuals (*P* < 0.001), reinforcing the plasma findings in the RCP cohort. Amyloid‐positive participants show elevated levels of CSF t‐tau and p‐tau181 (*P* < 0.001), reflecting neurodegeneration. CSF NfL levels are lower in amyloid‐positive participants, though the difference is not statistically significant (*P* > 0.05). Furthermore, plasma p‐tau217 levels were 3.6 to 5 times higher in amyloid‐positive patients than in amyloid‐negative patients in all cohorts. Detailed demographic, clinical, cognitive, genetic, and biomarker characteristics, stratified by cognitive status across all cohorts, are provided in Tables S3–S5 in supporting information.

### 3.2 Diagnostic accuracy of plasma biomarkers and ratios for amyloid status

**FIGURE 1 alz14536-fig-0001:**
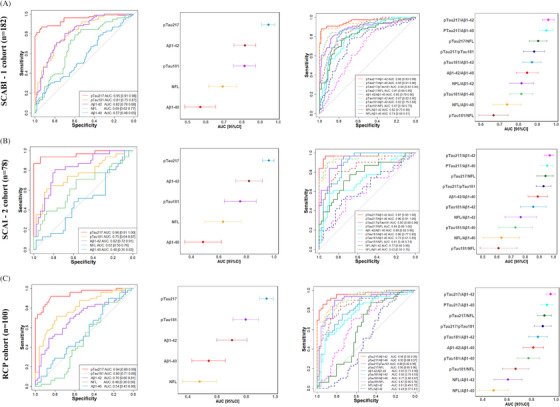
Diagnostic performance of plasma biomarkers across cohorts. ROC curves, AUC values, and diagnostic performance for plasma biomarkers in distinguishing amyloid‐positive from amyloid‐negative individuals in the SCABI‐1 (A), SCABI‐2 (B), and RCP (C) cohorts. Aβ, amyloid beta; AUC, area under the curve; CSF, cerebrospinal fluid; NfL, neurofilament light chain; p‐tau, phosphorylated tau; RCP, real‐world clinical practice; ROC, receiver operating characteristic; SCABI, Southern China Aging Brain Initiative.

Figure 1 illustrates the diagnostic performance (AUC) of plasma biomarkers and their ratios in distinguishing amyloid‐positive from amyloid‐negative participants across SCABI‐1, SCABI‐2, and RCP cohorts. Plasma p‐tau217 consistently demonstrates the highest diagnostic accuracy in all cohorts: SCABI‐1: AUC = 0.95 (95% CI: 0.91–0.98), SCABI‐2: AUC = 0.96 (95% CI: 0.91–1), RCP: AUC = 0.94 (95% CI: 0.89–0.99). Other biomarkers, such as Aβ1‐42 and p‐tau181, show moderate accuracy, with AUCs ranging from 0.70 to 0.82 depending on the cohort. NfL and Aβ1‐40 alone show relatively lower diagnostic accuracy (AUC < 0.70 in most cases), indicating limited standalone utility.

Ratios incorporating p‐tau217 significantly improve diagnostic performance; p‐tau217/Aβ1‐42 consistently achieves the highest AUC across cohorts: SCABI‐1: AUC = 0.96 (95% CI: 0.93–0.99), SCABI‐2: AUC = 0.97 (95% CI: 0.93–1), RCP: AUC = 0.96 (95% CI: 0.92–0.99). p‐tau217/Aβ1‐40 also demonstrates excellent performance, with AUC values comparable to p‐tau217/Aβ1‐42. Ratios involving p‐tau181 (e.g., p‐tau181/Aβ1‐42) show moderately strong performance but do not exceed those involving p‐tau217. Detailed performance values of single plasma biomarkers and their ratios across all cohorts are provided in Tables S6–S8 in supporting information.

### 3.3 Plasma p‐tau217 and p‐tau217/Aβ1‐42 show high diagnostic accuracy across research and clinical settings

**FIGURE 2:**
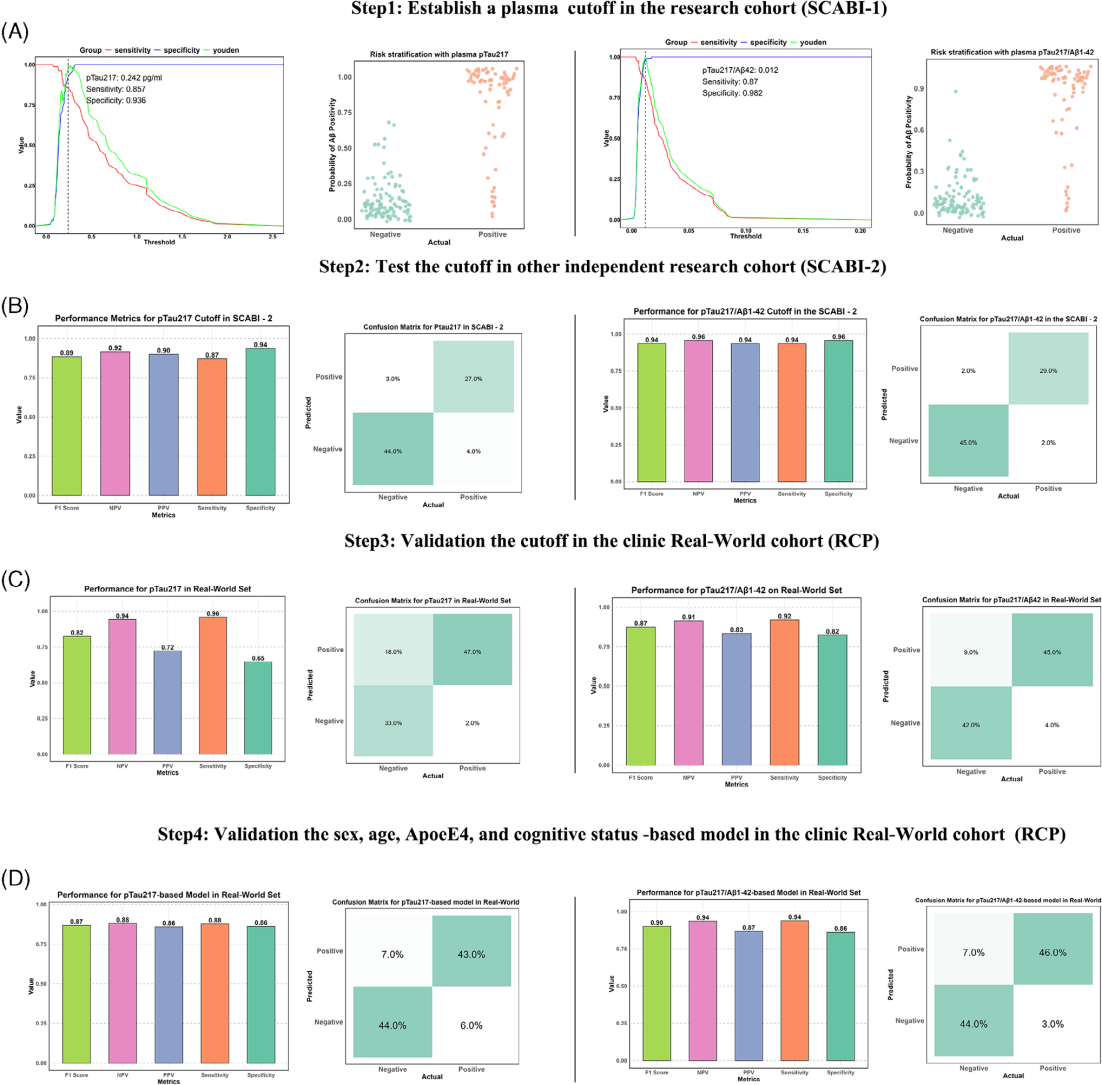
Stepwise validation of p‐tau217 and p‐tau217/Aβ1‐42 cutoffs. A, Establishment of optimal cutoffs in SCABI‐1 using the maximum Youden *J* index. B, Validation of the cutoffs in SCABI‐2 showing consistent performance metrics. C, Application of cutoffs to the real‐world RCP cohort, with a decrease in specificity observed. D, Integration of demographic and clinical factors (e.g., sex, age, *APOE* ε4 status, and cognitive status) in the RCP cohort, significantly improving diagnostic balance. Bar plots represent sensitivity, specificity, and F1 score, while confusion matrices detail classification accuracy. Aβ, amyloid beta; *APOE*, apolipoprotein E; AUC, area under the curve; CSF, cerebrospinal fluid; NfL, neurofilament light chain; NPV, negative predictive value; PPV, positive predictive value; p‐tau, phosphorylated tau; RCP, real‐world clinical practice; SCABI, Southern China Aging Brain Initiative.

To validate the applicability[Fig alz14536-fig-0001] of cutoff value in both research and clinical real‐world settings, Figure 2 illustrates the stepwise establishment and validation of plasma p‐tau217 and p‐tau217/Aβ1‐42 cutoffs for distinguishing amyloid‐positive from amyloid‐negative individuals across multiple cohorts.

Step 1 (Figure 2A), establishment of cutoffs in SCABI‐1: Plasma p‐tau217 and p‐tau217/Aβ1‐42 achieved high diagnostic accuracy, with cutoffs, sensitivity, and specificity values of 0.242 (pg/mL)/0.012, 0.86/0.87, and 0.94/0.98, respectively. Both biomarkers effectively stratified amyloid‐positive and amyloid‐negative participants. Step 2 (Figure 2B), validation of the cutoffs in SCABI‐2: In an independent cohort, both biomarkers maintained strong performance with p‐tau217: sensitivity = 0.87, specificity = 0.94 and p‐tau217/Aβ1‐42: sensitivity = 0.94, specificity = 0.96. Confusion matrices confirmed high agreement with true amyloid status, validating the cutoffs in a controlled research setting. Step 3 (Figure 2C), validation in the clinical real‐world setting: In the RCP cohort, p‐tau217 and p‐tau217/Aβ1‐42 showed strong diagnostic accuracy but slightly reduced specificity compared to research cohorts with p‐tau217: sensitivity = 0.96, specificity = 0.65 and p‐tau217/Aβ1‐42: sensitivity = 0.92, specificity = 0.82. The reduced specificity indicates challenges in correctly identifying amyloid‐negative individuals. Step 4 (Figure 2D), model‐based validation in RCP: Incorporating sex, age, *APOE* ε4, and cognitive status significantly improved diagnostic balance with p‐tau217: sensitivity = 0.88, specificity = 0.86 and p‐tau217/Aβ1‐42: sensitivity = 0.94, specificity = 0.86. The integration of demographic and clinical data mitigated the limitations observed in Step 3, improving specificity and overall diagnostic accuracy.

### 3.4 Correlations between plasma and CSF biomarkers

**FIGURE 3:**
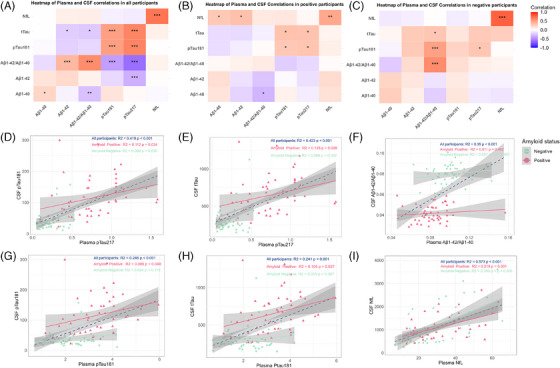
Scatterplots and correlations between CSF and plasma biomarkers in the RCP cohort. Heatmaps (A–C) illustrate correlations between plasma and CSF biomarkers across all participants (A), amyloid‐positive participants (B), and amyloid‐negative participants (C). D—I, Linear regression analyses between CSF and plasma biomarkers. Analyses are presented for all participants (green) and stratified by visual amyloid status (red: amyloid positive; blue: amyloid negative). Shaded areas indicate 95% confidence intervals for the regression lines. R‐squared (*R* ^2^) values and statistical significance (*P* values) are displayed in the upper‐right corner of each scatterplot. All biomarkers, except the amyloid ratio, are expressed in pg/mL. Aβ, amyloid beta; CSF, cerebrospinal fluid; NfL, neurofilament light chain; RCP, real‐world clinical practice; p‐tau, phosphorylated tau; t‐tau, total tau.

Plasma biomarkers, particularly p‐tau217, p‐tau181, and NfL, demonstrated strong correlations with their CSF counterparts across all participants in the RCP cohort, as shown in the heatmaps (Figure 3). Notably, plasma p‐tau217 and p‐tau181 exhibited robust correlations with CSF p‐tau181 and t‐tau (*R* ^2 ^> 0.4, *P* < 0.001), with stronger associations observed in amyloid‐positive participants. Similarly, plasma NfL correlated strongly with CSF NfL across all participants (*R* ^2^ = 0.57, *P* < 0.001), with particularly high concordance in amyloid‐negative individuals (*R* ^2^ = 0.70, *P* < 0.001). Plasma Aβ1‐42/Aβ1‐40 showed moderate correlations with CSF Aβ1‐42/Aβ1‐40 (*R* ^2^ = 0.35, *P* < 0.001), driven primarily by amyloid‐negative participants (*R* ^2^ = 0.60, *P* < 0.001). These results highlight the potential of plasma biomarkers, particularly p‐tau217, as reliable proxies for CSF measures, with diagnostic relevance across different amyloid statuses. Detailed correlation analyses, including additional scatterplots, are provided in Figure S1 in supporting information for further reference.

### 3.5 Correlations between plasma biomarker levels and amyloid PET values

**FIGURE 4:**
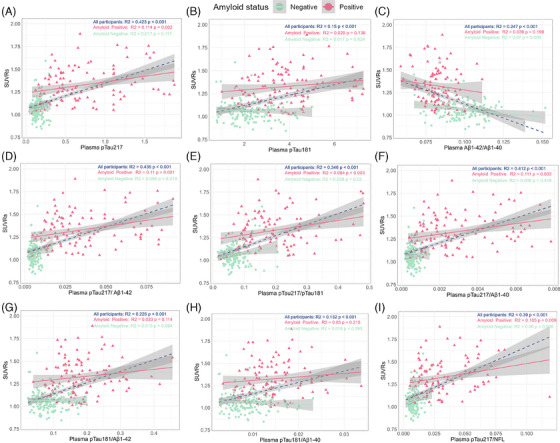
Scatterplots and correlations between PET SUVRs quantification and plasma biomarkers in the SCABI cohort. Scatterplots illustrating the linear regression analyses between PET SUVR values and plasma biomarkers, including p‐tau217, p‐tau181, NfL, Aβ1‐42, and their ratios. Data are presented for all participants (green), as well as stratified by amyloid visual status (red: amyloid positive; blue: amyloid negative). Shaded regions represent 95% confidence intervals for the regression lines. The R‐squared (*R* ^2^) values and statistical significance (*P* values) for each regression are displayed in the upper‐right corner of each plot. All biomarkers, except the amyloid ratio, are expressed in pg/mL. Aβ, amyloid beta; CSF, cerebrospinal fluid; PET, positron emission tomography; NfL, neurofilament light chain; p‐tau, phosphorylated tau; SCABI, Southern China Aging Brain Initiative; SUVR, standardized uptake value ratio; t‐tau, total tau.

Figure 4 illustrates the correlations between the levels of each plasma biomarker and PET amyloid deposition. Plasma biomarkers demonstrated significant correlations with SUVRs, particularly in amyloid‐positive individuals. Plasma p‐tau217 showed the strongest correlation across all participants (*R* ^2^ = 0.423, *P* < 0.001), with moderate associations in amyloid‐positive individuals (*R* ^2^ = 0.114, *P* = 0.002) but no significant correlation in amyloid‐negative individuals. Ratios involving p‐tau217, such as p‐tau217/Aβ1‐42, also correlated strongly with SUVRs (*R* ^2^ = 0.435, *P* < 0.001), with moderate associations in amyloid‐positive individuals (*R* ^2^ = 0.11, *P* = 0.001) and in amyloid‐negative individuals (*R* ^2^ = 0.089, *P* = 0.019). In contrast, plasma p‐tau181 and Aβ1‐42/Aβ1‐40 ratios showed weaker correlations with SUVRs (*R* ^2^ = 0.15 and *R* ^2^ = 0.247, respectively). These findings highlight the potential of p‐tau217 and their ratio as reliable plasma biomarkers for reflecting amyloid burden, particularly in amyloid‐positive individuals.

## 4 DISCUSSION

This study highlights the exceptional diagnostic performance of plasma p‐tau217 and p‐tau217/Aβ1‐42 in distinguishing amyloid‐positive from amyloid‐negative individuals across research and clinical settings. These biomarkers demonstrated high sensitivity and specificity in research cohorts, with real‐world validation showing improved diagnostic balance after adjusting for demographic and clinical factors. Their reliability and accessibility suggest a potential role in the early detection of AD, supporting timely diagnosis and enabling patient stratification for emerging therapies. To enhance AD management, plasma biomarkers should be integrated into a multimodal diagnostic approach, considering cognitive assessments, demographic characteristics, and other clinical evaluations to provide comprehensive care and avoid delays in diagnosis and treatment.

Among the individual biomarkers, plasma p‐tau217 demonstrated the highest accuracy in identifying brain amyloid status. Previous studies have assessed the performance of plasma biomarkers in detecting AD pathology using various analytical platforms. For instance, the AUC of plasma p‐tau217 ranged from 0.70 to 0.96,^7^, ^8^, ^17^, ^23^, ^24^, ^25^, ^26^ while that of plasma p‐tau181 varied from 0.70 to 0.9.^16^, ^27^, ^28^, ^29^ The Aβ1‐42/Aβ1‐40 ratio had an AUC ranging from 0.64 to 0.8.^9^, ^27^, ^30^, ^31^, ^32^ Other research has reported that plasma p‐tau217 can accurately detect brain pathology in both cognitively impaired^33^, ^34^ and cognitively healthy individuals.^24^ It can also distinguish AD from other neurodegenerative disorders^35^ and predict disease progression in preclinical and prodromal AD patients.^36^ Recent immunoassay research suggests that CSF p‐tau217 correlates better with amyloid PET and tau PET than CSF p‐tau181.^37^ Previous studies have used the Lumipulse G pTau 217 Plasma assay for detecting brain Aβ pathology, using CSF levels as the reference. These studies reported an AUC of 0.94,^17^, ^38^ which aligns with our findings. Our initial study used PET as a reference to evaluate the efficacy of the Lumipulse G plasma pTau 217 and other traditional AD biomarkers. In summary, whether CSF or PET was used as the reference, plasma p‐tau217 consistently demonstrated high accuracy; our findings support the utility of plasma p‐tau217 as a diagnostic tool in clinical applications.

Interestingly, the diagnostic balance of plasma biomarkers exhibited notable differences between the SCABI research cohort and the RCP real‐world clinical cohort. In the SCABI cohort, plasma biomarkers, particularly p‐tau217 and p‐tau217/Aβ1‐42, demonstrated consistently high sensitivity, specificity, and AUCs. These results reflect the controlled nature of the research setting, in which participants were more homogenous and amyloid status was determined using standardized PET imaging. In contrast, the real‐world RCP cohort, which included individuals with greater clinical heterogeneity, showed slightly reduced specificity for the biomarkers (p‐tau217: 65%, p‐tau217/Aβ1‐42: 82%), despite retaining high sensitivity (96% and 92%, respectively). This difference is likely attributable to the broader clinical population in the RCP cohort, characterized by greater comorbidities, variability in cognitive impairment severity, and amyloid status determination using CSF biomarkers rather than PET imaging. Mielke et al.^39^ also highlighted the impact of comorbidities, particularly kidney dysfunction, on plasma soluble tau. These methodological and population differences underscore the complexity of applying research‐derived cutoffs in routine clinical practice. To address these disparities, adjusting for demographic (age, sex, *APOE* ε4 status) and clinical (cognitive status) factors in the RCP cohort significantly improved specificity (both p‐tau217 and p‐tau217/Aβ1‐42: 86%) without compromising sensitivity. This adjustment highlights the critical role of tailoring diagnostic strategies to account for population‐specific characteristics, ensuring robust and reliable performance across diverse clinical settings. These findings emphasize the importance of validating research findings in real‐world contexts to bridge the gap between controlled research environments and routine clinical practice.

Our study reveals that plasma p‐tau217 levels were nearly 3.6‐ to 5‐fold greater in amyloid‐positive patients than in amyloid‐negative patients. Among the biomarkers, p‐tau217 showed the most significant changes and effect sizes, comparable to those in the CSF. This finding helps to clarify the uncertainty regarding cutoff values for plasma biomarkers and enhances the interpretability of clinical detection results. In previous studies, plasma p‐tau217 consistently demonstrated high accuracy across various platforms and strong correlations with AD markers such as traditional CSF biomarkers,^40^ amyloid PET findings,^41^ tau PET findings,^42^ and neuropathology.^43^ Notably, the fully automated platform used in our study simplifies the process, reduces manual intervention, enhances accessibility for clinical laboratories, and ensures greater reproducibility.^44^ These advantages highlight the potential of the automated plasma p‐tau217 assay for integration into routine clinical practice, though further validation in diverse populations and longitudinal studies remain essential.

Our study found that the plasma p‐tau217/Aβ1‐42 ratio accurately classified Aβ status (AUC 0.973). This assay has recently been shown to yield AUCs of up to 0.95 for p‐tau217/Aβ1‐42 in classifying CSF Aβ status,^17^ a finding that aligns with those obtained in the current PET study. Furthermore, we observed that ratios of p‐tau217 with Aβ1‐40, p‐tau181, and NfL had AUCs > 0.85. In contrast, the p‐tau181/Aβ1‐42 and Aβ1‐42/Aβ1‐40 ratios had AUCs > 0.80 in our study, indicating their efficacy in identifying brain amyloid status. A recent study suggested that the plasma p‐tau217/nop‐tau217 ratio was clinically equivalent to FDA‐approved CSF tests in classifying Aβ PET status, with AUCs of 0.95 to 0.97.^23^ Brum et al.^45^ proposed that combining plasma p‐tau217, age, and *APOE* status could improve the AUC to 0.89 to 0.94 in determining Aβ PET status. In recent research conducted with the Lumipulse platform, the plasma Aβ1‐42/Aβ1‐40 ratio achieved an AUC of 0.89, while p‐tau181 alone reached an AUC of 0.76 in detecting amyloid positivity.^10^ These results all suggest that composite measures of combined plasma biomarkers outperformed single biomarkers.^45^ This is likely due to the distinct biological information reflected by Aβ brain pathology and the reduced impact of higher plasma tau concentrations associated with low renal function.^46^ Therefore, we support the idea that the appropriate combination of biomarkers can enhance the accuracy and specificity of AD diagnosis.

In our study, we further combined the p‐tau217, p‐tau181, and NfL levels and the Aβ1‐42/Aβ1‐40 ratio to assess their joint predictive capability for brain amyloid status. This approach achieved an AUC of 0.96 and PPA, NPA, and overall percentage agreement values ranging from 0.90 to 0.95. However, compared to p‐tau217/Aβ1‐42 alone, combining multiple biomarkers did not significantly improve the ability to predict brain amyloid pathology. While these supplementary biomarkers contribute to the comprehensive evaluation of AD pathology, our findings underscore the need for a critical assessment of whether their inclusion provides substantial value over the robust diagnostic accuracy demonstrated by p‐tau217/Aβ1‐42 alone. Further research and rigorous comparative analyses are warranted to elucidate the incremental benefit of incorporating additional biomarkers into diagnostic algorithms, particularly in scenarios in which p‐tau217/Aβ1‐42 exhibits excellent performance alone.

The present study demonstrated strong correlations between plasma biomarkers and their CSF and PET counterparts, supporting their use as non‐invasive proxies for brain amyloid pathology. Plasma p‐tau217 showed the strongest correlations with CSF p‐tau181 and t‐tau (*R* ^2^ > 0.4, *P* < 0.001), particularly in amyloid‐positive individuals, consistent with previous studies linking p‐tau217 to tau and amyloid pathology.^47^, ^48^, ^49^ Plasma NfL also correlated strongly with CSF NfL (*R* ^2^ = 0.57, *P* < 0.001), especially in amyloid‐negative individuals (*R* ^2^ = 0.70), highlighting its relevance for detecting neurodegeneration.^50^ Plasma p‐tau217 and p‐tau217/Aβ1‐42 correlated significantly with amyloid PET SUVRs (*R* ^2^ = 0.42–0.44, *P* < 0.001), confirming their ability to quantify amyloid burden.^8^, ^42^ These findings reinforce the scalability of plasma biomarkers, though further validation across diverse populations remains critical for clinical implementation.

The inclusion of subjects from both research cohorts and clinical settings highlights the robustness of our study, minimizing selection bias and ensuring broad applicability. This approach minimizes selection bias and ensures a broad representation of research and clinical contexts. The stepwise validation—from establishing cutoffs in a research cohort to testing their applicability in independent research and real‐world clinical cohorts—ensures the robustness and generalizability of the findings. This methodology supports the translational relevance of plasma biomarkers for clinical practice. Furthermore, plasma p‐tau217 demonstrated high efficacy in predicting amyloid pathology within the Chinese demographic, representing nearly one fifth of the global population, using the Lumipulse G platform. Last, we have established plasma p‐tau217 as a reliable biomarker for categorizing patients based on their CSF and PET amyloid status.

Our study does have several limitations. First, it is a cross‐sectional study. Therefore, further validation in longitudinal studies is necessary to determine whether patients who test positive for plasma biomarkers will eventually progress to dementia. Second, while we considered the impact of disease complexity on plasma biomarkers, we did not specifically explore the influence of certain chronic conditions, such as chronic kidney disease, on plasma biomarker concentrations. However, previous studies have suggested that such conditions have a minimal impact on diagnostic performance.^46^ Moreover, it has been suggested that using ratios could help mitigate their effects.^39^ Third, the sample sizes for each cohort, particularly the RCP cohort, were relatively small, which may limit the statistical power and generalizability of the findings. Larger, multicenter studies are required to confirm these results. Finally, our study was conducted in a single center, which helped improve consistency. However, the accuracy and predictive ability of the cutoff values derived from our results must be validated using datasets from other institutions with similar characteristics.

In summary, our study demonstrates that plasma p‐tau217 and the p‐tau217/Aβ1‐42 ratio are highly accurate in detecting brain amyloid pathology across research and clinical settings. Incorporating demographic and clinical factors improves their diagnostic specificity in real‐world scenarios, supporting their use as accessible, non‐invasive tools for early AD diagnosis and patient stratification, bridging research findings with clinical application.

## CONFLICT OF INTEREST STATEMENT

Sen Liu is employed by, while Hong Zhang holds shares in, the Pason Neuroscience Research Center and Company. The remaining authors have declared that they have no conflicts of interest. Author disclosures are available in the supporting information.

## CONSENT STATEMENT

All participants or their legal guardians provided signed informed consent to participate in the study. The study adhered to the Declaration of Helsinki and was approved by the Affiliated Brain Hospital Ethics Committees of Guangzhou Medical University.

## Supporting information



Supporting Information

Supporting Information
