# Supplementary material for: Plasma p‐tau217 and p‐tau217/Aβ1‐42 are effective biomarkers for identifying CSF‐ and PET imaging‐diagnosed Alzheimer's disease: Insights for research and clinical practice
Source: Alzheimers Dement. 2025 Jan 30;21(2):e14536. doi: 10.1002/alz.14536 (PMC11848202; doi:10.1002/alz.14536)
Supplement: Supplementary file 1 — Supporting Information [file ALZ-21-e14536-s001.docx]

**Supplementary materials**

Supplementary Tables 2

**Table S1. the numbers (percentages) of participants with missing values in the SCABI cohort (n=260).** 2

**Table S2. the numbers (percentages) of participants with missing values in the RCP cohort (n=100).** 3

**Table S3. Clinical and demographic characteristics on cognitive status in the SCABI - 1 cohort (n=182).** 4

**Table S4. Clinical and demographic characteristics on cognitive status in the SCABI -2 cohort (n=78).** 8

**Table S5**  **Clinical and demographic characteristics on cognitive status in the RCP cohort (n=100).** 12

**Table S6 Performance values of single plasma biomarkers and their ratios in the SCABI - 1 cohort (n=182)** 15

**Table S7 Performance values of single plasma biomarkers and their ratios in the SCABI - 2 cohort (n=78)** 16

**Table S8 Performance values of single plasma biomarkers and their ratios in the RCP cohort (n=100)** 17

# **Supplementary Tables**

## **Table S1. the numbers (percentages) of participants with missing values in the SCABI cohort (n=260).**

|  | **SCABI cohort** | | | |
| --- | --- | --- | --- | --- |
|  | **Positive (n=107)** | | **Negative(n=153)** | |
|  | ***n*** | ***%*** | ***n*** | ***%*** |
| **Plasma biomarkers** |  |  |  |  |
| Aβ1-40(pg/ mL) | 1 | 0.93% | 1 | 0.65% |
| Aβ1-42(pg/ mL) | 0 | 0.00% | 0 | 0 |
| pTau181(pg/ mL) | 0 | 0.00% | 0 | 0 |
| pTau217(pg/ mL) | 0 | 0.00% | 0 | 0.00% |
| NfL(pg/ mL) | 11 | 10.30% | 10 | 6.54% |
| ApoE4 | 9 | 8.41% | 8 | 5.23% |
| **SUVRs** | 2 | 1.87 | 7 | 4.58% |

## **Table S2. the numbers (percentages) of participants with missing values in the RCP cohort (n=100).**

|  |  | **RCP cohort** | | |
| --- | --- | --- | --- | --- |
|  | **Negative(n=51)** | |  | **Positive(n=49)** |
|  | ***n*** | ***%*** | ***n*** | ***%*** |
| **Plasma biomarkers** | | | | |
| Aβ1-40(pg/ mL) | 0 | 0 | 0 | 0 |
| Aβ1-42(pg/ mL) | 0 | 0 | 0 | 0 |
| NfL(pg/ mL) | 1 | 1.96% | 3 | 6.12% |
| pTau181(pg/ mL) | 0 | 0 | 0 | 0 |
| pTau217(pg/ mL) | 2 | 3.92% | 2 | 4.08% |
| ApoE4 | 0 | 0 | 2 | 4.08% |
| **CSF biomarkers** | | | | |
| Aβ1-40(pg/ mL) | 0 | 0 | 0 | 0 |
| Aβ1-42(pg/ mL) | 0 | 0 | 0 | 0 |
| Aβ1-42/Aβ40 | 0 | 0 | 0 | 0 |
| tTau(pg/ mL) | 0 | 0 | 0 | 0 |
| pTau181(pg/ mL) | 0 | 0 | 0 | 0 |
| NfL(pg/ mL) | 0 | 0 | 0 | 0 |

## **Table S3.** **Clinical and demographic characteristics on cognitive status** **in the SCABI - 1 cohort (n=182).**

|  | **[ALL]** | **Cognitively Unimpaired** | **MCI** | **Dementia** | **p.overall** |
| --- | --- | --- | --- | --- | --- |
|  | ***N=182*** | ***N=36*** | ***N=120*** | ***N=26*** |  |
| Amyloid Status: |  |  |  |  | <0.001 |
| Negetive | 106 (58.2%) | 31 (86.1%) | 72 (60.0%) | 3 (11.5%) |  |
| Positive | 76 (41.8%) | 5 (13.9%) | 48 (40.0%) | 23 (88.5%) |  |
| Sex: |  |  |  |  | 0.139 |
| Male | 61 (33.5%) | 8 (22.2%) | 41 (34.2%) | 12 (46.2%) |  |
| Female | 121 (66.5%) | 28 (77.8%) | 79 (65.8%) | 14 (53.8%) |  |
| APOEe4: |  |  |  |  | 0.263 |
| Carrier | 47 (25.8%) | 6 (16.7%) | 32 (26.7%) | 9 (34.6%) |  |
| Non-Carrier | 135 (74.2%) | 30 (83.3%) | 88 (73.3%) | 17 (65.4%) |  |
| Age | 69.0 (7.03) | 67.9 (5.19) | 69.6 (6.89) | 67.9 (9.47) | 0.323 |
| Education | 10.1 (4.04) | 13.1 (3.74) | 9.69 (3.60) | 7.83 (4.20) | <0.001 |
| MMSE | 20.7 (6.41) | 25.9 (2.91) | 21.2 (5.17) | 11.1 (4.61) | <0.001 |
| ADL | 12.8 (2.38) | 13.9 (0.28) | 13.3 (1.84) | 9.23 (2.90) | <0.001 |
| HIS | 1.76 (0.98) | 1.72 (1.03) | 1.77 (0.96) | 1.77 (1.07) | 0.961 |
| CDR | 0.66 (0.50) | 0.38 (0.25) | 0.61 (0.40) | 1.29 (0.67) | <0.001 |
| Hypertension |  |  |  |  | 0.237 |
| with Hypertension | 62 (34.1%) | 9 (25.0%) | 46 (38.3%) | 7 (26.9%) |  |
| without Hypertension | 120 (65.9%) | 27 (75.0%) | 74 (61.7%) | 19 (73.1%) |  |
| Cardiology: |  |  |  |  | 0.310 |
| with Cardiology | 25 (13.7%) | 6 (16.7%) | 18 (15.0%) | 1 (3.85%) |  |
| without | 157 (86.3%) | 30 (83.3%) | 102 (85.0%) | 25 (96.2%) |  |
| Diabetes_mellitus: |  |  |  |  | 0.156 |
| with Diabetes_mellitus | 28 (15.4%) | 2 (5.56%) | 21 (17.5%) | 5 (19.2%) |  |
| without | 154 (84.6%) | 34 (94.4%) | 99 (82.5%) | 21 (80.8%) |  |
| Dyslipidemia: |  |  |  |  | 0.012 |
| with Dyslipidemia | 58 (31.9%) | 18 (50.0%) | 36 (30.0%) | 4 (15.4%) |  |
| without | 124 (68.1%) | 18 (50.0%) | 84 (70.0%) | 22 (84.6%) |  |
| **Plasma biomarkers** | | | | | |
| Aβ1-40(pg/ mL) | 272 (41.0) | 278 (28.4) | 269 (42.9) | 278 (46.8) | 0.397 |
| Aβ1-42(pg/ mL) | 24.6 (4.86) | 27.0 (4.35) | 24.2 (4.94) | 23.3 (4.20) | 0.003 |
| Aβ1-42/ Aβ1-40 | 0.09 (0.01) | 0.10 (0.01) | 0.09 (0.01) | 0.08 (0.01) | 0.001 |
| NFL(pg/ mL) | 21.6 (7.70) | 17.8 (6.53) | 21.2 (6.94) | 28.6 (8.24) | <0.001 |
| Ptau181(pg/ mL) | 2.98 (1.40) | 2.34 (1.08) | 2.91 (1.27) | 4.20 (1.63) | <0.001 |
| Ptau217(pg/ mL) | 0.38 (0.40) | 0.17 (0.11) | 0.31 (0.30) | 0.97 (0.52) | <0.001 |
| **Memory** | | | | | |
| AVLT_N13 | 14.2 (6.51) | 20.4 (6.34) | 13.1 (5.18) | 6.31 (3.47) | <0.001 |
| AVLT_N4 | 4.13 (3.23) | 7.14 (2.57) | 3.63 (2.87) | 0.38 (0.65) | <0.001 |
| AVLT_N5 | 3.52 (3.27) | 6.47 (2.86) | 2.98 (2.92) | 0.23 (0.44) | <0.001 |
| AVLT_N6 | 3.02 (3.30) | 6.47 (3.08) | 2.27 (2.73) | 0.15 (0.38) | <0.001 |
| **Language** | | | | | |
| BNT | 18.8 (4.61) | 23.0 (2.56) | 18.1 (4.23) | 13.8 (3.77) | <0.001 |
| VFT | 12.0 (4.23) | 15.4 (3.45) | 11.3 (3.83) | 7.58 (3.37) | <0.001 |
| **Executive function** | | | | | |
| TMTB | 85.8 (45.3) | 55.2 (23.1) | 91.5 (43.9) | 162 (41.2) | <0.001 |
| STROOP | 42.3 (7.13) | 46.7 (3.36) | 41.5 (7.31) | 34.2 (6.45) | <0.001 |
| **Attention** | | | | | |
| SDMT | 28.2 (13.5) | 39.5 (7.55) | 25.8 (12.6) | 12.9 (12.7) | <0.001 |
| DST | 14.8 (3.67) | 17.2 (3.33) | 14.4 (3.36) | 11.1 (3.20) | <0.001 |
| **Visuospatial skill** | | | | | |
| CDT | 3.04 (1.01) | 3.94 (0.23) | 2.93 (0.91) | 1.54 (0.97) | <0.001 |
| ROCF | 22.6 (8.24) | 29.0 (4.72) | 21.8 (7.45) | 12.4 (9.48) | <0.001 |
| **SUVRs** | 1.17 (0.19) | 1.12 (0.14) | 1.17 (0.21) | 1.25 (0.18) | 0.024 |

Abbreviations: MMSE, Mini-Mental State Examination; CDR, clinical dementia rating; ADL, activities of daily living; HIS, Hachinski Ischemic Score; AVLT N1-3, Auditory Verbal Learning Test Immediate recall; AVLT N4, Auditory Verbal Learning Test Short-term delayed recall; AVLT N5, Auditory Verbal Learning Test Long-term delayed recall; AVLT N6, Auditory Verbal Learning Test Recognition; BNT, Boston Naming Test; TMT, Trail-Making Test; ROCF, Rey-Osterrieth Complex; SDMT, Symbol-Digit Modality Test; SCWT, Stroop Color and Word Test; DST, Digit Span Test; CDT, Clock Drawing Test; CSF, cerebrospinal fluid; Aβ, amyloid β; pTau, phosphorylated tau; tTau, total tau; NfL, Neurofilament Light, SUVRs, Standardized Uptake Value Ratios

## **Table S4. Clinical and demographic characteristics on cognitive status in the SCABI -2 cohort (n=78).**

|  | **[ALL]** | **Cognitively Unimpaired** | **MCI** | **Dementia** | **p.overall** |
| --- | --- | --- | --- | --- | --- |
|  | ***N=78*** | ***N=18*** | ***N=41*** | ***N=19*** |  |
| Amyloid Status: |  |  |  |  | 0.002 |
| Negetive | 47 (60.3%) | 14 (77.8%) | 28 (68.3%) | 5 (26.3%) |  |
| Positive | 31 (39.7%) | 4 (22.2%) | 13 (31.7%) | 14 (73.7%) |  |
| Sex: |  |  |  |  | 0.504 |
| Male | 19 (24.4%) | 5 (27.8%) | 8 (19.5%) | 6 (31.6%) |  |
| Female | 59 (75.6%) | 13 (72.2%) | 33 (80.5%) | 13 (68.4%) |  |
| APOEe4: |  |  |  |  | 0.359 |
| Carrier | 23 (29.5%) | 4 (22.2%) | 11 (26.8%) | 8 (42.1%) |  |
| Non-Carrier | 55 (70.5%) | 14 (77.8%) | 30 (73.2%) | 11 (57.9%) |  |
| Age | 67.9 (7.58) | 67.1 (6.23) | 67.7 (7.63) | 69.0 (8.83) | 0.730 |
| Education | 9.77 (4.23) | 12.7 (3.22) | 9.54 (3.76) | 7.39 (4.64) | <0.001 |
| MMSE | 19.2 (6.39) | 24.9 (2.51) | 20.7 (3.96) | 10.6 (4.30) | <0.001 |
| ADL | 12.7 (2.36) | 13.9 (0.24) | 13.4 (1.43) | 10.3 (3.23) | <0.001 |
| HIS | 2.00 (1.08) | 2.28 (1.23) | 1.95 (1.09) | 1.84 (0.90) | 0.438 |
| CDR | 0.71 (0.54) | 0.42 (0.19) | 0.52 (0.16) | 1.39 (0.70) | <0.001 |
| Hypertension |  |  |  |  | 0.785 |
| with Hypertension | 27 (34.6%) | 5 (27.8%) | 15 (36.6%) | 7 (36.8%) |  |
| without Hypertension | 51 (65.4%) | 13 (72.2%) | 26 (63.4%) | 12 (63.2%) |  |
| Cardiology: |  |  |  |  | 0.333 |
| with Cardiology | 14 (17.9%) | 5 (27.8%) | 5 (12.2%) | 4 (21.1%) |  |
| without | 64 (82.1%) | 13 (72.2%) | 36 (87.8%) | 15 (78.9%) |  |
| Diabetes_mellitus: |  |  |  |  | 0.892 |
| with Diabetes_mellitus | 9 (11.5%) | 2 (11.1%) | 4 (9.76%) | 3 (15.8%) |  |
| without | 69 (88.5%) | 16 (88.9%) | 37 (90.2%) | 16 (84.2%) |  |
| Dyslipidemia: |  |  |  |  | 0.515 |
| with Dyslipidemia | 27 (34.6%) | 8 (44.4%) | 12 (29.3%) | 7 (36.8%) |  |
| without | 51 (65.4%) | 10 (55.6%) | 29 (70.7%) | 12 (63.2%) |  |
| **Plasma biomarkers** | | | | | |
| Aβ1-40(pg/ mL) | 263 (45.2) | 248 (49.8) | 269 (44.7) | 266 (40.5) | 0.272 |
| Aβ1-42(pg/ mL) | 24.7 (4.99) | 24.3 (4.24) | 25.1 (4.97) | 24.0 (5.77) | 0.663 |
| Aβ1-42/ Aβ1-40 | 0.09 (0.02) | 0.10 (0.02) | 0.09 (0.02) | 0.09 (0.01) | 0.220 |
| NFL(pg/ mL) | 20.4 (7.60) | 17.7 (6.59) | 19.8 (6.66) | 24.3 (9.14) | 0.022 |
| Ptau181(pg/ mL) | 2.84 (1.18) | 2.47 (1.25) | 2.64 (0.93) | 3.61 (1.31) | 0.003 |
| Ptau217(pg/ mL) | 0.39 (0.42) | 0.23 (0.26) | 0.35 (0.43) | 0.62 (0.46) | 0.014 |
| **Memory** | | | | | |
| AVLT_N13 | 13.8 (6.02) | 19.1 (5.88) | 12.5 (4.61) | 7.57 (3.41) | <0.001 |
| AVLT_N4 | 4.03 (3.11) | 6.78 (2.69) | 3.41 (2.56) | 0.57 (1.13) | <0.001 |
| AVLT_N5 | 3.61 (3.15) | 6.22 (2.88) | 3.00 (2.67) | 0.43 (1.13) | <0.001 |
| AVLT_N6 | 2.98 (3.03) | 5.94 (2.88) | 2.17 (2.31) | 0.14 (0.38) | <0.001 |
| **Language** | | | | | |
| BNT | 18.1 (4.50) | 22.3 (2.20) | 17.2 (3.78) | 12.1 (3.13) | <0.001 |
| VFT | 11.5 (3.80) | 14.7 (3.25) | 10.5 (3.16) | 7.83 (2.64) | <0.001 |
| **Executive function** | | | | | |
| TMTB | 95.9 (62.2) | 49.9 (11.1) | 108 (65.8) | 144 (51.8) | <0.001 |
| STROOP | 43.9 (6.40) | 46.2 (4.57) | 43.8 (5.77) | 36.4 (10.8) | 0.008 |
| **Attention** | | | | | |
| SDMT | 27.5 (14.3) | 43.3 (7.57) | 21.8 (11.5) | 16.5 (6.63) | <0.001 |
| DST | 14.1 (3.83) | 16.4 (2.59) | 13.2 (3.71) | 13.0 (5.18) | 0.008 |
| **Visuospatial skill** | | | | | |
| CDT | 3.03 (0.99) | 3.89 (0.32) | 2.78 (0.96) | 2.29 (0.95) | <0.001 |
| ROCF | 23.7 (7.50) | 29.3 (2.61) | 21.9 (7.84) | 19.1 (6.28) | <0.001 |
| **SUVRs** | 1.18 (0.21) | 1.09 (0.09) | 1.19 (0.23) | 1.25 (0.23) | 0.074 |

Abbreviations: MMSE, Mini-Mental State Examination; CDR, clinical dementia rating; ADL, activities of daily living; HIS, Hachinski Ischemic Score; AVLT N1-3, Auditory Verbal Learning Test Immediate recall; AVLT N4, Auditory Verbal Learning Test Short-term delayed recall; AVLT N5, Auditory Verbal Learning Test Long-term delayed recall; AVLT N6, Auditory Verbal Learning Test Recognition; BNT, Boston Naming Test; TMT, Trail-Making Test; ROCF, Rey-Osterrieth Complex; SDMT, Symbol-Digit Modality Test; SCWT, Stroop Color and Word Test; DST, Digit Span Test; CDT, Clock Drawing Test; CSF, cerebrospinal fluid; Aβ, amyloid β; pTau, phosphorylated tau; tTau, total tau; NfL, Neurofilament Light, SUVRs, Standardized Uptake Value Ratios.

## **Table S5** **Clinical and demographic characteristics on cognitive status in the RCP cohort (n=100).**

|  | **[ALL]** | **Cognitively Unimpaired** | **MCI** | **Dementia** | **p.overall** |
| --- | --- | --- | --- | --- | --- |
|  | ***N=100*** | ***N=1*** | ***N=28*** | ***N=71*** |  |
| Amyloid Status: |  |  |  |  | 0.035 |
| Negetive | 51 (51.0%) | 1 (100%) | 19 (67.9%) | 31 (43.7%) |  |
| Positive | 49 (49.0%) | 0 (0.00%) | 9 (32.1%) | 40 (56.3%) |  |
| Sex: |  |  |  |  | 0.056 |
| Male | 43 (43.0%) | 1 (100%) | 16 (57.1%) | 26 (36.6%) |  |
| Female | 57 (57.0%) | 0 (0.00%) | 12 (42.9%) | 45 (63.4%) |  |
| APOEe4: |  |  |  |  | 0.687 |
| Carrier | 38 (38.0%) | 0 (0.00%) | 9 (32.1%) | 29 (40.8%) |  |
| Non-Carrier | 62 (62.0%) | 1 (100%) | 19 (67.9%) | 42 (59.2%) |  |
| Age | 65.4 (10.9) | 73.0 (.) | 64.8 (8.47) | 65.5 (11.8) | 0.753 |
| Education | 8.65 (3.70) | 8.00 (.) | 8.74 (3.91) | 8.62 (3.67) | 0.976 |
| MMSE | 13.2 (7.66) | 25.0 (.) | 17.6 (7.40) | 10.9 (6.69) | <0.001 |
| ADL | 8.81 (4.56) | 14.0 (.) | 11.3 (3.70) | 7.58 (4.44) | 0.001 |
| HIS | 2.35 (1.58) | 1.00 (.) | 2.04 (1.04) | 2.52 (1.77) | 0.290 |
| CDR | 1.40 (0.87) | 0.50 (.) | 0.87 (0.64) | 1.65 (0.86) | <0.001 |
| Hypertension |  |  |  |  | 1.000 |
| with Hypertension | 27 (27.0%) | 0 (0.00%) | 8 (28.6%) | 19 (26.8%) |  |
| without Hypertension | 73 (73.0%) | 1 (100%) | 20 (71.4%) | 52 (73.2%) |  |
| Cardiology: |  |  |  |  | 0.748 |
| with Cardiology | 10 (10.0%) | 0 (0.00%) | 2 (7.14%) | 8 (11.3%) |  |
| without | 90 (90.0%) | 1 (100%) | 26 (92.9%) | 63 (88.7%) |  |
| Diabetes_mellitus: |  |  |  |  | 0.299 |
| with Diabetes_mellitus | 18 (18.0%) | 0 (0.00%) | 8 (28.6%) | 10 (14.1%) |  |
| without | 82 (82.0%) | 1 (100%) | 20 (71.4%) | 61 (85.9%) |  |
| Dyslipidemia: |  |  |  |  | 0.111 |
| with Dyslipidemia | 16 (16.0%) | 0 (0.00%) | 8 (28.6%) | 8 (11.3%) |  |
| without | 84 (84.0%) | 1 (100%) | 20 (71.4%) | 63 (88.7%) |  |
| **Plasma biomarkers** | | | | | |
| Aβ1-40(pg/ mL) | 269 (46.1) | 207 (.) | 283 (38.8) | 264 (47.7) | 0.076 |
| Aβ1-42(pg/ mL) | 24.4 (5.58) | 20.3 (.) | 25.7 (6.01) | 24.0 (5.39) | 0.313 |
| Aβ1-42/ Aβ1-40 | 0.09 (0.02) | 0.10 (.) | 0.09 (0.02) | 0.09 (0.02) | 0.899 |
| NFL(pg/ mL) | 31.6 (17.3) | 15.2 (.) | 27.1 (15.6) | 33.6 (17.7) | 0.159 |
| Ptau181(pg/ mL) | 2.74 (1.09) | 1.33 (.) | 2.31 (0.85) | 2.93 (1.12) | 0.015 |
| Ptau217(pg/ mL) | 0.48 (0.38) | 0.08 (.) | 0.35 (0.35) | 0.54 (0.38) | 0.046 |
| **CSF biomarkers** | | | | | |
| Aβ1-40(pg/ mL) | 5993 (2935) | 14167 (.) | 6213 (2629) | 5790 (2916) | 0.015 |
| Aβ1-42(pg/ mL) | 361 (242) | 1430 (.) | 397 (185) | 332 (227) | <0.001 |
| Aβ1-42/ Aβ1-40 | 0.06 (0.02) | 0.10 (.) | 0.07 (0.02) | 0.06 (0.02) | 0.116 |
| pTau181(pg/ mL) | 75.7 (61.4) | 57.6 (.) | 63.0 (63.0) | 81.0 (60.8) | 0.407 |
| tTau(pg/ mL) | 479 (273) | 417 (.) | 400 (257) | 511 (276) | 0.187 |
| NfL(pg/ mL) | 1334 (1266) | 1050 (.) | 1030 (1173) | 1457 (1298) | 0.315 |
| **Memory** | | | | | |
| AVLT_N13 | 8.17 (5.10) | 10.0 (.) | 8.31 (5.66) | 8.00 (4.77) | 0.915 |
| AVLT_N4 | 1.29 (1.86) | 2.00 (.) | 1.58 (2.39) | 1.03 (1.31) | 0.508 |
| AVLT_N5 | 0.92 (1.60) | 3.00 (.) | 1.23 (2.01) | 0.59 (1.10) | 0.135 |
| AVLT_N6 | 0.92 (2.02) | 1.00 (.) | 1.46 (2.75) | 0.44 (0.89) | 0.204 |
| **Language** | | | | | |
| BNT | 15.5 (5.36) | 23.0 (.) | 16.4 (5.32) | 14.6 (5.26) | 0.162 |
| VFT | 9.02 (3.38) | 12.0 (.) | 9.00 (3.25) | 8.90 (3.63) | 0.680 |
| **Executive function** | | | | | |
| TMTB | 111 (67.4) | 76.0 (.) | 93.3 (46.4) | 134 (83.5) | 0.176 |
| STROOP | 37.8 (10.2) | 46.0 (.) | 38.2 (11.7) | 36.7 (8.67) | 0.673 |
| **Attention** | | | | | |
| SDMT | 21.1 (13.1) | 37.0 (.) | 21.3 (13.9) | 19.9 (12.2) | 0.459 |
| DST | 13.0 (3.72) | 12.0 (.) | 12.8 (3.66) | 13.3 (3.94) | 0.854 |
| **Visuospatial skill** | | | | | |
| CDT | 2.41 (1.24) | 4.00 (.) | 2.83 (1.13) | 2.00 (1.20) | 0.019 |
| ROCF | 15.2 (10.5) | 23.5 (.) | 16.3 (10.9) | 13.9 (10.4) | 0.522 |

Abbreviations: MMSE, Mini-Mental State Examination; CDR, clinical dementia rating; ADL, activities of daily living; HIS, Hachinski Ischemic Score; AVLT N1-3, Auditory Verbal Learning Test Immediate recall; AVLT N4, Auditory Verbal Learning Test Short-term delayed recall; AVLT N5, Auditory Verbal Learning Test Long-term delayed recall; AVLT N6, Auditory Verbal Learning Test Recognition; BNT, Boston Naming Test; TMT, Trail-Making Test; ROCF, Rey-Osterrieth Complex; SDMT, Symbol-Digit Modality Test; SCWT, Stroop Color and Word Test; DST, Digit Span Test; CDT, Clock Drawing Test; CSF, cerebrospinal fluid; Aβ, amyloid β; pTau, phosphorylated tau; tTau, total tau; NfL, Neurofilament Light, SUVRs, Standardized Uptake Value Ratios.

## **Table S6 Performance values of single plasma biomarkers and their ratios in the SCABI - 1 cohort (n=182)**

|  | AUC (95%CI) | Sensitivity (95%CI) | Specificity (95%CI) | PPV | NPV | OPA | Cutoff (95%CI) | VR |
| --- | --- | --- | --- | --- | --- | --- | --- | --- |
| pTau217 | 0.946 (0.909, 0.982) | 0.868 (0.774, 0.927) | 0.934 (0.870, 0.968) | 0.904 | 0.908 | 0.907 | 0.242 (0.215, 0.290) | 0.900 |
| pTau181 | 0.814 (0.753, 0.875) | 0.711 (0.600, 0.800) | 0.783 (0.695, 0.851) | 0.701 | 0.790 | 0.753 | 3.135 (2.815, 3.560) | 0.746 |
| Aβ1-42 | 0.816 (0.755, 0.876) | 0.803 (0.700, 0.877) | 0.698 (0.605, 0.777) | 0.656 | 0.831 | 0.742 | 24.447 (23.565, 25.770) | 0.749 |
| Aβ1-40 | 0.569 (0.483, 0.654) | 0.289 (0.200, 0.400) | 0.858 (0.780, 0.912) | 0.595 | 0.628 | 0.621 | 241.000 (230.035, 253.920) | 0.498 |
| NFL | 0.693 (0.616, 0.771) | 0.789 (0.685, 0.866) | 0.594 (0.499, 0.683) | 0.583 | 0.797 | 0.676 | 18.600 (17.735, 22.180) | 0.685 |
| pTau217/Aβ1-42 | 0.959 (0.927, 0.991) | 0.882 (0.790, 0.936) | 0.981 (0.934, 0.995) | 0.971 | 0.920 | 0.940 | 0.012 (0.010, 0.017) | 0.93 |
| PTau217/Aβ1-40 | 0.948 (0.912, 0.984) | 0.868 (0.774, 0.927) | 0.981 (0.934, 0.995) | 0.971 | 0.912 | 0.934 | 0.001 (0.001, 0.001) | 0.923 |
| pTau217/pTau181 | 0.883 (0.828, 0.939) | 0.803 (0.700, 0.877) | 0.868 (0.790, 0.920) | 0.813 | 0.860 | 0.841 | 0.101 (0.090, 0.115) | 0.835 |
| pTau217/NFL | 0.905 (0.858, 0.952) | 0.829 (0.729, 0.897) | 0.896 (0.824, 0.941) | 0.851 | 0.880 | 0.868 | 0.012 (0.011, 0.014) | 0.862 |
| Aβ1-42/Aβ1-40 | 0.846 (0.788, 0.905) | 0.855 (0.759, 0.917) | 0.774 (0.685, 0.843) | 0.730 | 0.882 | 0.808 | 0.089 (0.086, 0.095) | 0.813 |
| pTau181/Aβ1-42 | 0.872 (0.822, 0.921) | 0.763 (0.656, 0.845) | 0.811 (0.726, 0.874) | 0.744 | 0.827 | 0.791 | 0.132 (0.111, 0.146) | 0.787 |
| pTau181/Aβ1-40 | 0.815 (0.754, 0.876) | 0.803 (0.700, 0.877) | 0.726 (0.635, 0.802) | 0.678 | 0.837 | 0.758 | 0.010 (0.009, 0.012) | 0.764 |
| pTau181/NFL | 0.667 (0.588, 0.745) | 0.842 (0.744, 0.907) | 0.472 (0.379, 0.566) | 0.533 | 0.806 | 0.626 | 0.111 (0.100, 0.132) | 0.63 |
| NFL/Aβ1-40 | 0.739 (0.664, 0.814) | 0.776 (0.671, 0.855) | 0.689 (0.595, 0.769) | 0.641 | 0.811 | 0.725 | 0.075 (0.069, 0.086) | 0.731 |
| NFL/Aβ1-42 | 0.816 (0.753, 0.880) | 0.763 (0.656, 0.845) | 0.774 (0.685, 0.843) | 0.707 | 0.820 | 0.769 | 0.916 (0.832, 1.029) | 0.768 |

Abbreviations: AUC, areas under the curve; PPA, positive percent agreement; NPA, negative percent agreement; OPA, overall percent agreement; Aβ, amyloid β; pTau, phosphorylated tau; tTau, total tau; NfL, Neurofilament Light, VR, Value Retention.

## **Table S7** **Performance values of single plasma biomarkers and their ratios in the SCABI - 2 cohort (n=78)**

|  | AUC (95%CI) | Sensitivity | Specificity | PPV | NPV | OPA | Cutoff | VR |
| --- | --- | --- | --- | --- | --- | --- | --- | --- |
| pTau217 | 0.962 (0.914, 1.000) | 0.935 (0.793, 0.982) | 0.936 (0.828, 0.978) | 0.906 | 0.957 | 0.936 | 0.227 (0.200, 0.256) | 0.935 |
| pTau181 | 0.755 (0.639, 0.871) | 0.613 (0.438, 0.763) | 0.872 (0.748, 0.940) | 0.760 | 0.774 | 0.769 | 3.250 (2.885, 4.095) | 0.731 |
| Aβ1-42 | 0.818 (0.722, 0.914) | 0.806 (0.637, 0.908) | 0.787 (0.651, 0.880) | 0.714 | 0.860 | 0.795 | 24.615 (23.885, 28.170) | 0.796 |
| Aβ1-40 | 0.485 (0.351, 0.618) | 1.000 (0.890, 1.000) | 0.085 (0.034, 0.199) | 0.419 | 1.000 | 0.449 | 194.840 (179.935, 218.130) | 0.292 |
| NFL | 0.630 (0.498, 0.763) | 0.710 (0.534, 0.839) | 0.574 (0.433, 0.705) | 0.524 | 0.750 | 0.628 | 18.680 (17.385, 22.640) | 0.638 |
| pTau217/Aβ1-42 | 0.974 (0.934, 1.000) | 0.968 (0.838, 0.994) | 0.936 (0.828, 0.978) | 0.909 | 0.978 | 0.949 | 0.009 (0.007, 0.013) | 0.952 |
| PTau217/Aβ1-40 | 0.956 (0.908, 1.000) | 0.968 (0.838, 0.994) | 0.851 (0.723, 0.926) | 0.811 | 0.976 | 0.897 | 0.001 (0.001, 0.001) | 0.908 |
| pTau217/pTau181 | 0.930 (0.878, 0.982) | 0.839 (0.674, 0.929) | 0.894 (0.774, 0.954) | 0.839 | 0.894 | 0.872 | 0.112 (0.101, 0.149) | 0.866 |
| pTau217/NFL | 0.944 (0.883, 1.000) | 0.903 (0.751, 0.967) | 0.915 (0.801, 0.966) | 0.875 | 0.935 | 0.910 | 0.012 (0.010, 0.015) | 0.909 |
| Aβ1-42/Aβ1-40 | 0.889 (0.820, 0.959) | 0.968 (0.838, 0.994) | 0.681 (0.538, 0.796) | 0.667 | 0.970 | 0.795 | 0.096 (0.091, 0.097) | 0.812 |
| pTau181/Aβ1-42 | 0.857 (0.766, 0.948) | 0.806 (0.637, 0.908) | 0.809 (0.675, 0.896) | 0.735 | 0.864 | 0.808 | 0.107 (0.100, 0.145) | 0.807 |
| pTau181/Aβ1-40 | 0.731 (0.612, 0.850) | 0.581 (0.408, 0.736) | 0.894 (0.774, 0.954) | 0.783 | 0.764 | 0.769 | 0.013 (0.011, 0.017) | 0.721 |
| pTau181/NFL | 0.611 (0.480, 0.742) | 0.516 (0.348, 0.680) | 0.723 (0.582, 0.831) | 0.552 | 0.694 | 0.641 | 0.154 (0.140, 0.199) | 0.611 |
| NFL/Aβ1-40 | 0.631 (0.501, 0.761) | 0.516 (0.348, 0.680) | 0.766 (0.628, 0.864) | 0.593 | 0.706 | 0.667 | 0.085 (0.079, 0.111) | 0.629 |
| NFL/Aβ1-42 | 0.768 (0.657, 0.879) | 0.774 (0.602, 0.886) | 0.681 (0.538, 0.796) | 0.615 | 0.821 | 0.718 | 0.754 (0.697, 0.958) | 0.726 |

Abbreviations: AUC, areas under the curve; PPA, positive percent agreement; NPA, negative percent agreement; OPA, overall percent agreement; Aβ, amyloid β; pTau, phosphorylated tau; tTau, total tau; NfL, Neurofilament Light, VR, Value Retention.

## **Table S8 Performance values of single plasma biomarkers and their ratios in the RCP cohort (n=100)**

|  | AUC (95%CI) | Sensitivity | Specificity | PPV | NPV | OPA | Cutoff | VR |
| --- | --- | --- | --- | --- | --- | --- | --- | --- |
| pTau217 | 0.939 (0.892, 0.986) | 0.918 (0.808, 0.968) | 0.843 (0.720, 0.918) | 0.849 | 0.915 | 0.880 | 0.318 (0.284, 0.394) | 0.880 |
| pTau181 | 0.797 (0.709, 0.884) | 0.714 (0.576, 0.822) | 0.784 (0.654, 0.875) | 0.761 | 0.741 | 0.750 | 2.828 (2.565, 3.130) | 0.748 |
| Aβ1-42 | 0.702 (0.598, 0.806) | 0.490 (0.356, 0.625) | 0.863 (0.743, 0.932) | 0.774 | 0.638 | 0.680 | 20.870 (20.055, 23.555) | 0.650 |
| Aβ1-40 | 0.543 (0.428, 0.657) | 0.898 (0.782, 0.956) | 0.255 (0.155, 0.389) | 0.537 | 0.722 | 0.570 | 229.185 (219.265, 250.185) | 0.479 |
| NFL | 0.480 (0.363, 0.596) | 0.837 (0.710, 0.915) | 0.314 (0.203, 0.450) | 0.539 | 0.667 | 0.570 | 42.340 (39.795, 53.580) | 0.513 |
| pTau217/Aβ1-42 | 0.956 (0.917, 0.995) | 0.898 (0.782, 0.956) | 0.902 (0.790, 0.957) | 0.898 | 0.902 | 0.900 | 0.014 (0.011, 0.017) | 0.900 |
| PTau217/Aβ1-40 | 0.926 (0.877, 0.975) | 0.837 (0.710, 0.915) | 0.863 (0.743, 0.932) | 0.854 | 0.846 | 0.850 | 0.001 (0.001, 0.002) | 0.850 |
| pTau217/pTau181 | 0.891 (0.822, 0.960) | 0.898 (0.782, 0.956) | 0.824 (0.697, 0.904) | 0.830 | 0.894 | 0.860 | 0.146 (0.116, 0.185) | 0.860 |
| pTau217/NFL | 0.905 (0.848, 0.962) | 0.878 (0.758, 0.943) | 0.784 (0.654, 0.875) | 0.796 | 0.870 | 0.830 | 0.011 (0.009, 0.014) | 0.830 |
| Aβ1-42/Aβ1-40 | 0.813 (0.729, 0.898) | 0.755 (0.619, 0.854) | 0.745 (0.611, 0.845) | 0.740 | 0.760 | 0.750 | 0.091 (0.087, 0.097) | 0.750 |
| pTau181/Aβ1-42 | 0.852 (0.776, 0.929) | 0.837 (0.710, 0.915) | 0.765 (0.632, 0.860) | 0.774 | 0.830 | 0.800 | 0.101 (0.093, 0.122) | 0.800 |
| pTau181/Aβ1-40 | 0.774 (0.681, 0.867) | 0.551 (0.413, 0.681) | 0.902 (0.790, 0.957) | 0.844 | 0.676 | 0.730 | 0.012 (0.011, 0.015) | 0.705 |
| pTau181/NFL | 0.669 (0.562, 0.777) | 0.714 (0.576, 0.822) | 0.627 (0.490, 0.747) | 0.648 | 0.696 | 0.670 | 0.090 (0.077, 0.132) | 0.669 |
| NFL/Aβ1-40 | 0.490 (0.372, 0.608) | 0.837 (0.710, 0.915) | 0.333 (0.220, 0.470) | 0.547 | 0.680 | 0.580 | 0.162 (0.132, 0.192) | 0.528 |
| NFL/Aβ1-42 | 0.606 (0.492, 0.721) | 0.878 (0.758, 0.943) | 0.451 (0.323, 0.586) | 0.606 | 0.793 | 0.660 | 0.831 (0.731, 1.056) | 0.629 |

Abbreviations: AUC, areas under the curve; PPA, positive percent agreement; NPA, negative percent agreement; OPA, overall percent agreement; Aβ, amyloid β; pTau, phosphorylated tau; tTau, total tau; NfL, Neurofilament Light, VR, Value Retention.

# **Supplementary Figure**


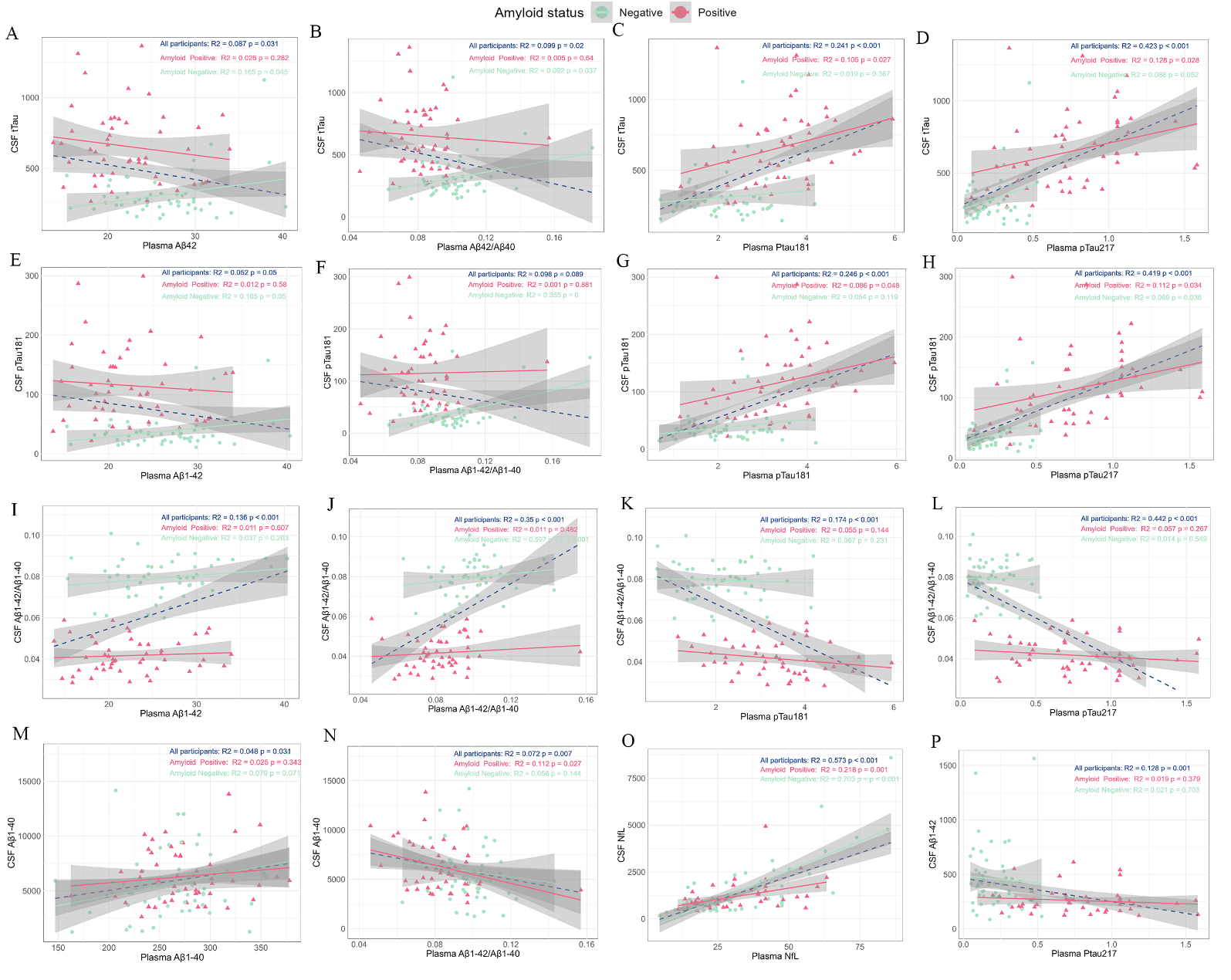


## **Supplementary Figure. Correlations between plasma and CSF biomarkers stratified by amyloid status.**

Panels (A–P) illustrate the correlations between plasma biomarkers (x-axis) and their corresponding CSF biomarkers (y-axis), stratified by amyloid status (positive vs. negative). Scatterplots display data points for amyloid-positive (red triangles) and amyloid-negative (green circles) participants, with regression lines and R^2^ values for each group as well as the combined cohort. Biomarkers analyzed include Aβ1-42, Aβ1-42/Aβ1-40, pTau181, pTau217, and NfL. Results highlight strong correlations between plasma and CSF biomarkers, particularly for amyloid-positive individuals, reinforcing the potential utility of plasma biomarkers in reflecting CSF biomarker levels across amyloid statuses.

Abbreviations: AUC, areas under the curve; PPA, positive percent agreement; NPA, negative percent agreement; OPA, overall percent agreement; Aβ, amyloid β; pTau, phosphorylated tau; tTau, total tau; NfL, Neurofilament Light.
